# Congenital Amaurosis Spontaneously Cured on the First Appearance of the Mestrual Function

**Published:** 1880-08

**Authors:** 


Congenital Amaurosis Spontaneously Cured on the First Appearance of the Mestrual Function.—A lady of twenty-two years of age consulted Dr. Santos Fernandes. She is of good form, nervous, lymphatic temperament, and was born blind. Her parents having lost all hope of having her sight restored, she became resigned to her fate. When about fourteen years of age, the girl one morning was able to see a little, the objects being dimly visible. Being much astounded, she returned to bed, and her mother found her clothes bloody. Her neighbors flocked to see the girl restored to sight. When presented to Fernandes her sight was perfect. Whether a similar case can result in a similar manner remains to be seen.—*Revita Medecina Cirurgia,* Madrid, No. 84.

